# 

**Published:** 1924-06

**Authors:** 


### ANSWERS TO CORRESPONDENTS.

COLLECTOR.—Statistics showing the results of street collections can be obtained from the Advisory Committee on Street Collections, New Scotland Yard, S.W.

ENQUIRER.—Messrs. George Jennings, of 67 Lambeth Palace Road, S.E. 1, specialise in hospital sanitary equipment, and would probably have just what you want.

IN MEMORY.—Advisers in connection with Hospital Tablets and Memorials are:—The Church Crafts League, Church House, Westminster, S.W. 1, The Civic Arts Association, 20 Buckingham Street, Strand, W.C. 2, English Crafts and Monumental Society, 1a Kensington Place, W. 8.

C. G.—Possibly the Clapham Maternity Hospital, Jeffreys Road, Clapham, S.W., might take the case. Payment is by small weekly contributions, according to means.

PIPING HOT.—Your difficulty with regard to keeping food hot could easily be overcome by the use either of a water-heated trolley such as those supplied by Sumerling & Co., Ltd., 63 Bunhill Row, E.C., or an electrically heated trolley which could be got from Benham & Sons, Ltd., 64 Wigmore Street, W. 1.

Miss S.—The National Library for the Blind is at 18 Tufton Street, Westminster, S.W. 1. The secretary is Miss O. I. Prince.

### Opening of Lady Reading's Hospital, Simla.

Lady Reading's Hospital for Indian Women and Children has been formally opened at Simla. It supplements the scheme originally begun by Lady Dufferin, and her hospital, now outgrown, becomes the out-patient department and welfare centre. The staff of the new institution consists of two English women doctors, two English nursing sisters and Indian nurses and probationers. Perhaps the most interesting part of the scheme is its provision for training Indian nurses. The generous response to Lady Reading's appeal has enabled the hospital to be not only equipped but endowed.

### RECENT APPOINTMENTS.

### Medical Officers, &c.

Cullen, C. K., M.R.C.S., L.R.C.P., Second Assistant M.O. and Deputy Medical Superintendent, High Wood Hospital for Children, Brentwood; Assistant Tuberculosis Officer, Poplar.

Faill, Charles James Campbell, L.R.C.S., L.R.C.P., Tuberculosis Officer for Bristol; Chief Clinical Tuberculosis Officer, Leeds (£900).

Jacobs, Cyril, M.C., M.B., B.S.; Ophthalmic Surgeon, Ebbw Vale, Education Committee (Durham University).

Morley, D. E., M.R.C.S., L.R.C.P.; Second Assistant M.O.H., Bath.

Shelley, H. M., M.R.C.S., L.R.C.P.; Government M.O., Nyasaland.

### Public Health and Hospital Appointments.

Allen, J., C.M.B.; Superintendent, Mother and Child Welfare Centre, Campden Hill, Kensington.

Bamber, Mary, C.M.B.; Health Visitor, Burnley.

Baugh, Winifred Mary, Health Visitor, Monmouthshire County Council; Health Visitor, Cardiff.

Beardwell, N., School Nurse and Health Visitor, Leigh.

Beattie, J., Health Visitor, Liverpool; Health Nurse, Birkenhead.

Breeze, Mildred, Matron, Brookfield Orthopædic Hospital.

Brown, Jessie S.; Matron, Hospital for Tuberculosis, Langstone.

Cotton, A., Sister, Hope Hospital, Pendleton; Health Nurse, Birkenhead.

Davis, Gladys Kate; Health Visitor and School Nurse, Rhondda.

Dewer, Isabella, Superintendent (Queen Victoria's Jubilee Institute, Scotland), Ayrshire.

Griffiths, Edith Grace, Tuberculosis Nurse, Deptford Metropolitan Borough.

Grogan, E., Health Visitor, Liverpool; Health Nurse, Birkenhead.

Jasper, Mildred, C.D.B., Health Visitor, Wolverhampton.

Johnson, G. M., C.M.B., Royal United Hospital, Bath; Matron, London Female Lock Hospital, Harrow Road.

Kaneen, I., Sister, Birkenhead Infirmary; Health Nurse, Birkenhead.

Knowles, E. J., Matron, Manchester Sanatorium, Abergele.

Lees, Lucy; Matron, Cottage Hospital, Ramsbottom.

MacDonald, Lilian, Tuberculosis Health Visitor, Plymouth.

Morris, Agnes M.; Matron, Cottage Hospital, Chirk.

Phillips, A. M.; Matron, Children's Tuberculosis Sanatorium, Honeylands, Exeter.

Rawes, Alice; Matron, War Pensions After Care Home, Dunston-on-Tyne, Northumberland.

Sansom, Ruth, Tuberculosis Health Visitor, Plymouth.

Thomas, Eva; Matron, West Bromwich and District Hospital.

Turner, Thomas, A.R.S.I.; District Sanitary Inspector, Stoke-on-Trent.

Warner, Florence Jane, C.M.B.; Matron, Barnwood Second County Medical Hospital, Coney Hill.

Webster, Mary, R.R.C. (Second Class); Matron, Prince Edward Memorial Hospital, Rhyl.

Wickenden, Percy; Honorary Secretary, Eye and Ear Hospital, Tunbridge Wells.

Williams, C. B., Health Visitor, Llanberis; Health Nurse, Birkenhead.

Young, Emily W., C.M.B., Assistant Matron.

### Town Planning.

The Minister of Health is often asked for a brief statement of the general scope and object of town-planning schemes and of the procedure to be followed in preparing them. To meet this need the Ministry has issued a fourpenny booklet on "Town Planning in England and Wales," which can be obtained from H.M. Stationery Office.

### Managerial Notices.

*All letters on Business matters must be addressed to the Manager, "The Hospital and Health Review," 28 and 29 Southampton Street, London, W.C. 2.*

RATES FOR SUBSCRIPTION (payable in advance).

| | | | |
|---|---|---|---|
| Three Months (including Postage) | .. .. | 1s. | 9d. |
| Six Months do. | .. .. | 3s. | 6d. |
| Twelve Months do. | .. .. | 7s. | 0d. |

**It is especially requested that remittances be made by Cheque or Postal Order and not Stamps.**

Subscriptions, which may commence at any time, are payable in advance. Cheques and Post-Office Orders should be crossed Westminster Bank, Covent Garden Branch, and made payable to the Manager of THE HOSPITAL AND HEALTH REVIEW.

***Advertisements.*** Scale of charges and particulars of special positions may be obtained on application to the Manager.

*SUBSCRIPTION FORM.*

*Please send me* THE HOSPITAL AND HEALTH REVIEW *for............ months, commencing with your issue of ............, for which please find enclosed ........*

*Name* ..........................................

*Address* ..........................................

..........................................

*Date* ..........................................

*To*.......................................*Newsagent*

